# Effects of Anti-Integrin Treatment With Vedolizumab on Immune Pathways and Cytokines in Inflammatory Bowel Diseases

**DOI:** 10.3389/fimmu.2018.01700

**Published:** 2018-07-31

**Authors:** Timo Rath, Ulrike Billmeier, Fulvia Ferrazzi, Michael Vieth, Arif Ekici, Markus F. Neurath, Raja Atreya

**Affiliations:** ^1^Department of Medicine 1, Division of Gastroenterology, Pneumology and Endocrinology, Ludwig Demling Endoscopy Center of Excellence, University of Erlangen-Nuremberg, Erlangen, Germany; ^2^Institute of Human Genetics, Friedrich-Alexander-Universität Erlangen-Nuremberg, Erlangen, Germany; ^3^Institute of Pathology, Klinikum Bayreuth, Bayreuth, Germany

**Keywords:** inflammatory bowel diseases, ulcerative colitis, Crohn’s disease, vedolizumab, RNA sequencing, integrin, cytokines

## Abstract

**Background and aims:**

Despite proven clinical efficacy of vedolizumab (VDZ) for inducing and maintaining remission in patients with Crohn’s disease (CD) and ulcerative colitis (UC), subgroups of patients have no therapeutic benefit from anti-α4β7 integrin therapy with VDZ. Within this study, we aimed to identify genetic, cellular, and immunological mechanisms that define response and failure to VDZ treatment.

**Methods:**

Intestinal RNA sequencing was performed in UC and CD patients before and at week 14 of VDZ therapy. α4β7 expression on peripheral and mucosal immune cells was assessed by flow cytometry and immunohistochemistry. Cellular modes of VDZ-mediated action were analyzed *ex vivo* and in VDZ-treated inflammatory bowel disease patients.

**Results:**

Transcriptome analysis showed an impairment of signaling cascades associated with adhesion, diapedesis, and migration of granulocytes and agranulocytes upon VDZ therapy. In non-remitters to VDZ therapy, a tissue destructive and leukocyte-mediated inflammatory activity with activation of TNF-dependent pathways was present, all of which were inhibited in remitters to VDZ. Clinical remission was associated with a significant reduction of α4β7 expression on Th2 and Th17 polarized mucosal CD4^+^ T cells at week 14 of VDZ therapy and with significantly higher numbers of α4β7-expressing mucosal cells prior to the initiation of VDZ therapy compared with non-remitters.

**Conclusion:**

Intestinal α4β7 expression prior to VDZ therapy might represent a biomarker that predicts therapeutic response to subsequent VDZ treatment. Due to high activation of TNF signaling in VDZ non-remitters, anti-TNF treatment might represent a promising therapeutic strategy in VDZ refractory patients.

## Introduction

Rapid recruitment of leukocytes from the blood stream into the intestinal lamina propria is a key process for the homeostatic immune surveillance and the exaggerated mucosal immune response that is observed in inflammatory bowel diseases (IBD) such as Crohn’s disease (CD) and ulcerative colitis (UC). The process of leukocyte migration into the mucosa is a multistep cascade involving tethering, rolling, adhesion, and finally diapedesis of leukocytes, all of which is orchestrated by a complex network of cell-intrinsic and -extrinsic factors as well as regulatory molecules on T cells and endothelial cells, such as PSGL-1, ICAM-1, and LFA-1 and in addition by chemokines and their specific receptors (1, 2). Since marked lymphocyte accumulation within the lamina propria is one of the pathogenic and histologic hallmarks observed in IBD patients, targeting T cell homing to the gut has emerged as a novel and promising therapeutic option in IBD patients.

This is particularly relevant for the integrin α4β7 that is expressed on the surface of gut-tropic effector lymphocytes. Homing of α4β7-expressing T cells is mediated by the specific interaction between α4β7 and its ligand mucosal addressin cell adhesion molecule-1 (MAdCAM-1), which is expressed under steady-state conditions by gut endothelial cells (3). Experimental models of chronic intestinal inflammation using a murine antibody against the α4β7 integrin have shown that the inhibition of the interaction between MAdCAM-1 and α4β7 can successfully inhibit the entry of CD4^+^ T cells to the intestinal mucosa system and efficiently suppress intestinal inflammation (4, 5). Based on this experimental evidence, vedolizumab (VDZ) was developed as a humanized antibody that binds to a conformational epitope that is unique to the heterodimerization of the human α4 with the β7 chain (6, 7), thereby conferring specificity toward the α4β7 integrin. Subsequently, VDZ was shown to suppress migration and accumulation of α4β7-bearing effector T cells from UC patients to the inflamed colon *in vivo* suggesting that this antibody may suppress lymphocyte trafficking in IBD (8, 9). Clinical studies revealed that VDZ has statistically significant therapeutic efficacy in placebo controlled phase 3 clinical trials of patients with moderately-to-severe active UC (10) or CD leading to the approval of VDZ in the US and Europe for the treatment of both IBD entities (11, 12).

Nevertheless, only a distinct proportion of 40–60% of IBD patients will achieve clinical response under VDZ therapy (11, 12), and only 15% of CD patients will achieve clinical remission at week 10 of treatment (12, 13). Furthermore, in light of the increasing diversification of biological therapy modalities (14, 15) and to allow a targeted, timely, effective, and economic treatment, factors that define or predict therapeutic response are needed in daily practice. Based on these considerations, we aimed to define genetic and immunological differences between responders and non-responders to VDZ therapy and to identify factors that predict therapeutic response prior to the initiation of anti-adhesion molecule therapy with VDZ.

## Materials and Methods

### Clinical Disease Activity and Definition of Response and Remission

During VDZ induction therapy, patients were seen at week 0, 2, 6, 10, and 14 in the IBD outpatient department. At each visit, clinical scoring was assessed using the Harvey–Bradshaw index (HBI) for CD (16) and the Mayo Clinic Score for UC (17, 18). At week 14, therapeutic response toward VDZ was assessed. Clinical remission was defined as a HBI < 5 for CD (19) and as a Mayo Clinic score of 2 or lower and no subscore higher than 1 for UC (11). Clinical response was defined as a decrease from baseline in Mayo score by ≥30% and ≥3 points, with a decrease in rectal bleeding subscore ≥ 1 or rectal bleeding subscore of 0 or 1 (UC) or a decrease in HBI > 4 points from baseline (CD).

### IBD Patients and Flow Cytometric Analysis of Human Blood and Lamina Propria Mononuclear Cells

To determine the α4β7 expression on subsets of T cells in humans, peripheral blood and colonic samples from IBD patients were analyzed. Human intestinal lamina propria cells and peripheral blood mononuclear cells (PBMCs) from IBD and control patients were isolated as previously described (20, 21). After gating on CD3^+^CD4^+^ T cells, the following signature cytokines and transcription factors were used with the following antibodies to analyze differentially polarized T cells: Th1: IFNγ (anti-human IFNγ APC, eBiosciences, Cat. 17-7319-82); Tbet (anti-human/mouse T-bet PerCPCy5.5, eBiosciences, Cat. 45-5825-82), Th2: IL-4 (anti-human IL-4 APC, eBiosciences, Cat. 17-7049-82), IL-13 (anti-human IL-13 PerCP/Cy5.5, BioLegend, Cat. 501911), GATA3 (anti-human/mouse Gata-3 PE, eBiosciences, Cat. 12-9966-42); Th17: IL-17 (anti-human IL-17A PerCP-Cy5.5, Cat. 45-7179-42).

α4β7 was visualized using commercially available VDZ (Takeda, Tokyo, Japan), which was stably labeled with fluorescein isothiocyanate (FITC). For analysis of α4β7 expression on peripheral blood lymphocytes under VDZ therapy, 12 patients with CD and 10 patients with UC were included. From all patients, blood samples were obtained before the initiation of anti-integrin therapy with VDZ and directly before the second, third, and fourth administration of 300 mg VDZ by intravenous infusion at weeks 0, 2, 6, and 14 respectively (11). For analysis of α4β7 expression on lamina propria mononuclear cells, a total of 11 patients with UC and 12 patients with CD were included. Of the 11 patients with UC, 5 had a clinical remission while 6 UC patients were refractory to VDZ therapy. Of the twelve patients with CD, six patients were remitters while six CD patients did not have remission to VDZ therapy and α4β7 expression was comparatively assessed between remitters and non-remitters.

The study was carried out in accordance with the recommendations of the ethical committee of the University Hospital, Friedrich-Alexander-Universität Erlangen-Nürnberg, Germany. The protocol was approved by the ethical committee of the University Hospital, Friedrich-Alexander-Universität Erlangen-Nürnberg, Germany. Each patient gave written informed consent in accordance with the Declaration of Helsinki before inclusion into the study.

### Expression of Th1, Th2, and Th17 Polarizing Cytokines From PBMCs and Regulation of Cell Death in the Presence of VDZ

Peripheral blood mononuclear cells from IBD patients were isolated and cultured in RPMI medium 1640 (Gibco) containing 10% FCS (Pan Biotech), and 1% penicillin/streptomycin. Cells were stimulated with anti-human CD3 (eBioscience, clone OKT3) and anti-human CD28 (BD Pharmingen, clone CD28.2) at a final concentration of 1 µg/mL in the presence of 40 µg/mL VDZ or IgG1 isotype control. After 24 and 48 h, supernatant was collected, and quantification of cytokine production was performed by ELISA for IFN-γ (human IFNγ ELISA Ready-SET-Go, eBiosciences, Cat. 88-7316), IL-4 (Human IL-4 ELISA Ready-SET-Go, eBiosciences, Cat. 88-7046), IL-17a (human IL-17A ELISA Ready-SET-Go, eBiosciences, Cat. 88-7176), and IL-10 (BD OptEIA Human IL-10 ELISA Kit II, BD Pharmingen, Cat. 550613). In separate experiments, PBMCs from IBD patients (UC: *n* = 7; CD: *n* = 7) were cultured for 24 and 48 h and induction of cell death in the presence of 40 µg/mL VDZ or IgG1 isotype control was assessed using the cell death detection plus Kit (Roche Diagnostics, Mannheim, Germany).

### Effects of VDZ and Isotype Control on the Adhesion of PBMCs to MAdCAM-1 Expressing HeLa Cells

Expression of MAdCAM-1 on HeLa cells was verified by immunohistochemistry using rabbit anti-human MAdCAM-1 (anti-MAdCAM, Abcam, Cat. ab178549) or an isotpye control antibody at 1 µg/mL as primary antibodies and anti-rabbit Alexa-647 as a secondary antibody. For adhesion assays, MAdCAM-1 expressing HeLa cells were grown to confluence for 48 to 72 h in μ-Slides (μ-Slide 8 Well, Ibidi, Cat. 80826) Subsequently, 5 × 10^5^ PBMCs labeled with a vital dye from healthy controls (*n* = 4) were preincubated with 40 µg/mL VDZ or IgG1 isotype control for 60 min and then added to HeLa cells. After 60 min, non-attaching PBMCs were washed of by three vigorous washes with TBS. Cells were fixed with 4% paraformaldehyde and HeLa cells were stained for MAdCAM-1 as described above followed by nuclear counterstaining with DAPI before final analyses by fluorescence microscopy (Keyence, Osaka, Japan).

In separate experiments, cryopreserved PBMCs from VDZ naïve IBD patients (UC: *n* = 3; CD: *n* = 3) and matched PBMC controls from the same patients after VDZ induction therapy were thawn, stained with vital dye, and added to confluently grown HeLa cells for 60 min. Afterward, non-attaching cells were similarly washed off and were fixed with 4% paraformaldehyde. HeLa cells were stained for MAdCAM-1 as described above followed by nuclear counterstaining with DAPI before final analyses by fluorescence microscopy (Keyence Corp., BZ-X710).

### Internalization of α4β7 Over Time in the Presence of VDZ

1 × 10^6^ PBMCs from each of seven IBD patients were isolated and cultured for the indicated time points in the presence of 40 µg/mL VDZ or IgG1 isotype control. Afterward, cells were stained for surface expression of CD3^+^, CD4^+^, and CD8^+^ for 15 min at room temperature. Subsequently, half of the cells were permeabilized before α4β7 staining with FITC-labeled VDZ, while in the other α4β7 staining with FITC-VDZ was performed prior to permeabilization of the cells. Mean fluorescence intensity (MFI) of the FITC signal in the respective CD3, CD4, and CD8 gates were analyzed by flow cytometry.

In another set of experiments, PBMCs or peripheral CD4^+^ T cells isolated from IBD patients using magnetic sorting (CD4 Microbeads, MACS, Miltenyi, Cat. No 130-045-101) were cultured in the presence of 40 µg/mL FITC-labeled VDZ or FITC-labeled IgG1 isotype control for 24 h. Cells were then fixed in 4% paraformaldehyde and after nuclear counterstaining with DAPI, internalization of FITC-VDZ or FITC-IgG1 was assessed by confocal microscopy (Leica TCS SP8, Leica, Germany).

### Immunohistochemistry of Mucosal α4β7 Expression in IBD Patients

Cryosections from colonic biopsies of IBD patients were used for immunohistochemistry. Tissue sections were fixed in 4% paraformaldehyde, followed by sequential incubation with avidin/biotin- (Vector Laboratories), and protein-blocking reagent (Roth) to suppress unspecific background staining. Sections were incubated with primary antibodies specific for human CD3 (rat anti-human CD3, Bio-Rad, Cat. MCA1477), human CD4 (rat anti-human CD4, Bio-Rad, Cat. MCA484G), and FITC-labeled vedolizumab (Entyvio^®^, Takeda). Furthermore, sections were incubated with an isotype matched control antibody as negative controls. Subsequently, samples were incubated with Alexa 647 conjugated secondary antibodies. Nuclei were counterstained with DAPI before final analysis by confocal microscopy (Leica SP8 Microscope). Positive cells in 6–10 high power fields (HPFs) were subsequently counted in all patients. In some images, an inset of a higher magnification was included to better identify the stained nuclei.

### RNA Sequencing Analysis

Total RNA was extracted from biopsy specimens using the RNeasy Mini Kit according to the manufacturer’s instructions. Quality of RNA was analyzed using a 2100 Bioanalyzer system (Agilent Technologies). RNA sequencing was performed in the Next-Generation-Sequencing Core Unit of the University of Erlangen-Nürnberg. Library preparation, sequencing on a HighSeq-2500 platform (Illumina, San Diego, CA, USA), as well as mapping and counting of reads were performed as previously described (22).

Sequencing data analyses were performed using R version 3.2.1 (R Foundation for Statistical Computing, Vienna, Austria) and Bioconductor (23), separately for CD and UC samples. In particular, differential expression analysis of genes with non-zero median count was performed with the DESeq2 package v.1.8.1 (24). First, expression before vs. after therapy was compared, controlling for patient effects. Afterward, the response to therapy was included in the design to analyze expression in responders and non-responders. Genes with *p*-value <0.01 were considered to be differentially expressed. Heatmaps of differentially expressed genes were obtained using for each gene regularized log-transformed data standardized across samples. Functional annotation analysis was performed using Ingenuity Pathway Analysis (IPA, QIAGEN Redwood City). A network-level visualization of the relationships between enriched pathways identified by IPA was obtained relying on Cytoscape (25). Similarity between a pair of pathways was taken equal to the Jaccard similarity coefficient calculated between the associated gene sets. Only pathways with *p*-value <0.025 and similarity >0.3 with at least one other pathway were visualized.

### Statistical Analysis

Statistics were processed using SPSS (SPSS Inc., Chicago, IL, USA) and GraphPad Prism (GraphPad Software, La Jolla, CA, USA). Values presented in this manuscript represent mean ± SEM. For statistical analyses, after testing for normal distribution, significant differences between samples were calculated using non-parametric tests (Mann–Whitney *U* test). A two-tailed *p* ≤ 0.05 was considered statistical significant: *p ≤ 0.05; **p ≤ 0.01; ***p ≤ 0.001.

## Results

### RNA Sequencing of Responders and Non-Responders Before and After VDZ Induction Therapy

To gain large-scale insights into underlying differences between remitters and non-remitters to anti-integrin therapy with VDZ, we initially performed whole transcriptome expression analysis using an RNA sequencing approach. For this purpose, we analyzed mucosal gene expression in 10 IBD patients both before and at week 14 of VDZ therapy. Sequencing yielded a median of 47 M reads per sample, with 58% median counted reads. The analyzed cohorts were comprised of three CD and two UC patients with remission upon VDZ therapy (remitters), as well as three CD and two UC patients in which VDZ failed to induce clinical remission (non-remitters). For all patients, biopsies obtained before commencement of VDZ and at week 14 of VDZ therapy were analyzed, thereby allowing control for patient-specific variability.

**Figure 1:**
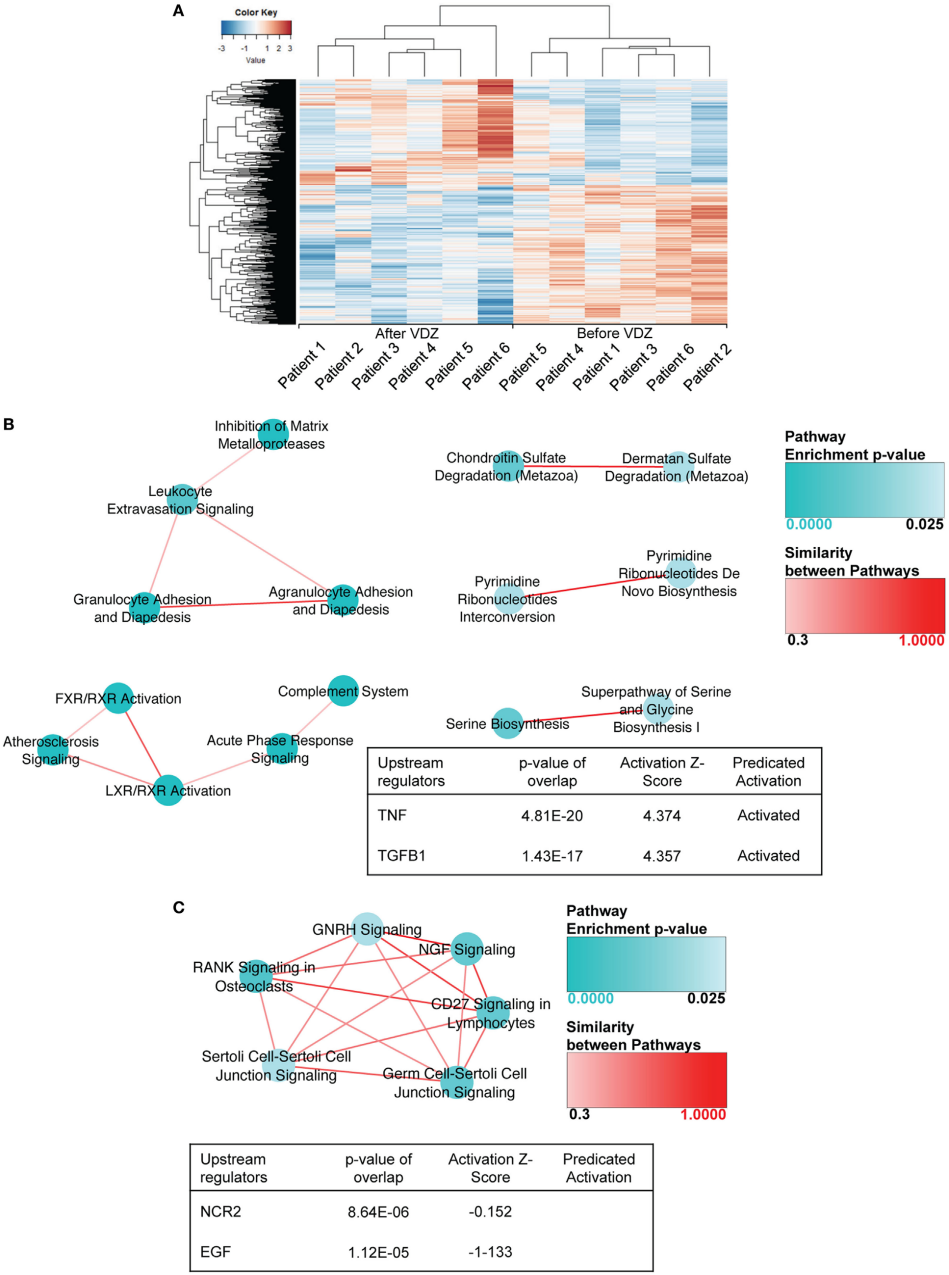
RNA sequencing in patients with Crohn’s disease under treatment with anti-α4β7 therapy with vedolizumab (VDZ). **(A)** Unsupervised hierarchical clustering of the expression profiles of differentially expressed genes showed two distinct clusters of expression, discriminating Crohn’s disease (CD) patients before and after VDZ therapy. **(B)** Canonical pathways and upstream regulators associated with differentially expressed genes in CD patients with no remission to VDZ therapy. In non-remitters, among the 1,014 genes that were differentially regulated in biopsies obtained after completed VDZ therapy compared with matched biopsies obtained prior to VDZ induction, many contained pro-inflammatory properties such as members of the TNF signaling cascade, various pro-inflammatory cyto- and chemokines or its receptors, and several members of the metalloproteinase family. In CD patients with no remission to VDZ therapy, granulocyte adhesion and diapedesis were the most significantly regulated canonical pathway and TNF as well as TGFβ1 were the most significantly regulated upstream regulators and both were predicted to be activated. **(C)** Canonical pathways and upstream regulators associated with differentially expressed genes in CD patients with remission to VDZ therapy. In VDZ remitters, RANK signaling and CD27 signaling in lymphocytes were among the most significantly regulated pathways and NCR2 and EGF were the most significantly regulated upstream regulators. Compared with non-remitters the pro-inflammatory signature was considerably less present, and among all genes significantly regulated by at least 1.5-fold, only three contained immunogenic and pro-inflammatory properties. Turquoise dots represent pathway enrichment *p*-values; similarity of the pathways is represented by red lines.

Given the high differences expected between CD and UC, biopsy samples for the two diseases were analyzed separately. In a first set of analyses, differences in the composition of the transcriptome before and after VDZ induction therapy in CD and UC patients were analyzed. To identify which genes were differentially expressed, we relied on the DESeq2 package within the R/Bioconductor environment. This analysis identified a total of 883 differentially expressed genes in CD (*p*-value <0.01), with 382 genes showing an increased expression and 501 genes showing a decreased expression at week 14 of VDZ therapy when compared with before initiation of VDZ therapy (Table S1 in Supplementary Material). As shown in Figure 1A, unsupervised hierarchical clustering of the expression profiles of differentially expressed genes showed clear discrimination between CD patients before and after completion of VDZ induction therapy. Interestingly, when analyzing underlying signaling pathways in the differentially expressed genes in CD with IPA program, both granulocyte adhesion and diapedesis as well as agranulocyte adhesion and diapedesis were among the top three canonical pathways identified (Table S2 in Supplementary Material), thereby not only corroborating the proclaimed mode of action of VDZ at the transcriptional level but also extending these mechanisms to agranulocytic cells.

To better understand differences in the composition of the transcriptome between remitters and non-remitters to VDZ therapy on RNA level, we comparatively assessed canonical pathways as well as upstream regulators that were differentially regulated between CD patients with remission and those without remission upon VDZ therapy, with disease remission defined *via* clinical disease activity scoring (HBI < 5) (19). Baseline disease and clinical characteristics before and after VDZ induction therapy are comparatively shown for CD patients with remission to VDZ induction therapy and those exhibiting no remission toward VDZ in Table 1. No significant confounder regarding therapeutic response could be found in this comparative listing.

**Table 1:** Clinical data of Crohn’s disease (CD) patients included for RNA sequencing.

	CD			
	Remitters (*n* = 3)		Non-remitters (*n* = 3)	
**Baseline characteristics**				

Sex (m/f)	0/3		2/1	
Age (years)				
Mean ± SD	38.6 ± 19.5		44.9 ± 5.2	
Disease manifestation				
Ileum				
Ileum + colon	1		2	
Ileum + colon + upper GI tract	2		1	
Disease duration				
Mean ± SD (years)	6.7 ± 1.6		7.3 ± 4.5	
Prior anti-TNF treatment	3		3	
Concomittant medication				
Glucocorticoids only	1		1	
Immunosuppressants only^a^			1	
No glucocorticoids and immunosuppressants	2		1	

	**Before vedolizumab (VDZ)**	**VDZ week 14**	**Before VDZ**	**VDZ week 14**

Disease activity, CD				
HBI, mean ± SD^b^	14 ± 1.2	2.3 ± 1	14.3 ± 1.5	12 ± 6
SES-CD, mean ± SD^c^	15.6 ± 3.2	7.6 ± 2.1	5.6 ± 4.9	6.3 ± 5.5
Leukocyte count ± SD (×10^9^/L)	9.5 ± 2.4	10.6 ± 6.4	7.3 ± 1	7.2 ± 1.1
CrP ± SD (mg/L, reference < 5)	8.3 ± 10.9	10.3 ± 11	6.2 ± 9.8	4.5 ± 4.9

*^a^Immunosuppressants included azathioprine, methotrexate, and mercaptopurine*.

*^b^The Harvey–Bradshaw index (HBI) ranges from 0 to 26, with higher scores indicating more active disease. Disease remission was defined as a HBI < 5*.

*^c^The Simple Endoscopic Score for Crohn’s Disease (SES-CD) assesses ulcer size, ulcerated surface, surface affected from disease and the presence of stenosis and ranges from 0 to 56, with higher scores indicating more severe disease*.

When analyzing the transcriptional expression profile in three CD patients with no remission upon VDZ therapy, we found a total of 1,014 genes to be differentially regulated (*p* < 0.01) in biopsies obtained at week 14 of VDZ therapy compared with matched biopsies form the same colonic segment obtained prior to VDZ induction (Table S3 in Supplementary Material). Importantly, among those genes significantly upregulated by at least 1.5-fold, many contained pro-inflammatory properties such as members of the TNF signaling cascade (*TNI3P, TNFAIP6, TNFRSF10C, TNFSF8, TNFSF15*, and *TNFSF13B)* and various pro-inflammatory cyto- and chemokines or its corresponding receptors (*IL31RA, IL23A, IL7R, IRAK2, IL33*, and *CXCL6*). Of note, many of the chemo- and cytokines found to be upregulated in CD patients with no remission upon VDZ have been previously shown to be involved in the generation of pathogenic Th2 and Th17 responses such as IL-33 and IL-31 (26–29) and furthermore, the IL-33/ST2 axis (30–35), IL-31 (36), as well as IL-23 (37–39) all of which have been previously implicated in the pathogenesis of T cell-mediated intestinal inflammation. Furthermore, several members of the metalloproteinase family were upregulated (*MME, MMP3, MMP10, ADAM12, MMP2, MMP7*, and *MMP12*), indicative of the presence of a tissue destructive milieu in CD patients with no remission to VDZ. When analyzing underlying signaling pathways of the differentially expressed genes in non-remitters to therapy, granulocyte adhesion and diapedesis were the most significantly regulated canonical pathway and TNF as well as TGFβ1 were the most significantly regulated upstream regulators with a predicted activation of both cytokines (Figure 1B). Altogether, these analyses were suggestive of a persistent level of leukocyte-mediated inflammatory activity that is driven by TNF as a major pro-inflammatory cytokine and TGFβ1, the latter of which has been associated with stricturing CD (40).

When analyzing the transcriptome in CD patients with remission to VDZ therapy, 263 genes were differentially expressed (*p* < 0.01) and the pro-inflammatory signature was considerably less present: among those genes significantly upregulated by at least 1.5-fold, only three contained immunogenic and pro-inflammatory properties (*CXCL13, TGFβ2*, and *TNFSF11*) and no tissue destructive signature was observed in CD patients with remission to VDZ therapy (Table S4 in Supplementary Material). On IPA, RANK signaling and CD27 signaling in lymphocytes were among the most significantly regulated pathways and NCR2 and EGF were the most significantly regulated upstream regulators (Figure 1C). Hence, while TNF-driven inflammatory activity was observed in non-remitters to VDZ, remitters to therapy showed no clear pro-immunogenic milieu consistent with the idea that non-remitters are characterized by TNF-mediated mucosal inflammation. Our findings thus suggest that these patients may benefit from therapeutic strategies that target alternative pathways to leukocyte migration, especially anti-TNF targeted treatment.

**Figure 2:**
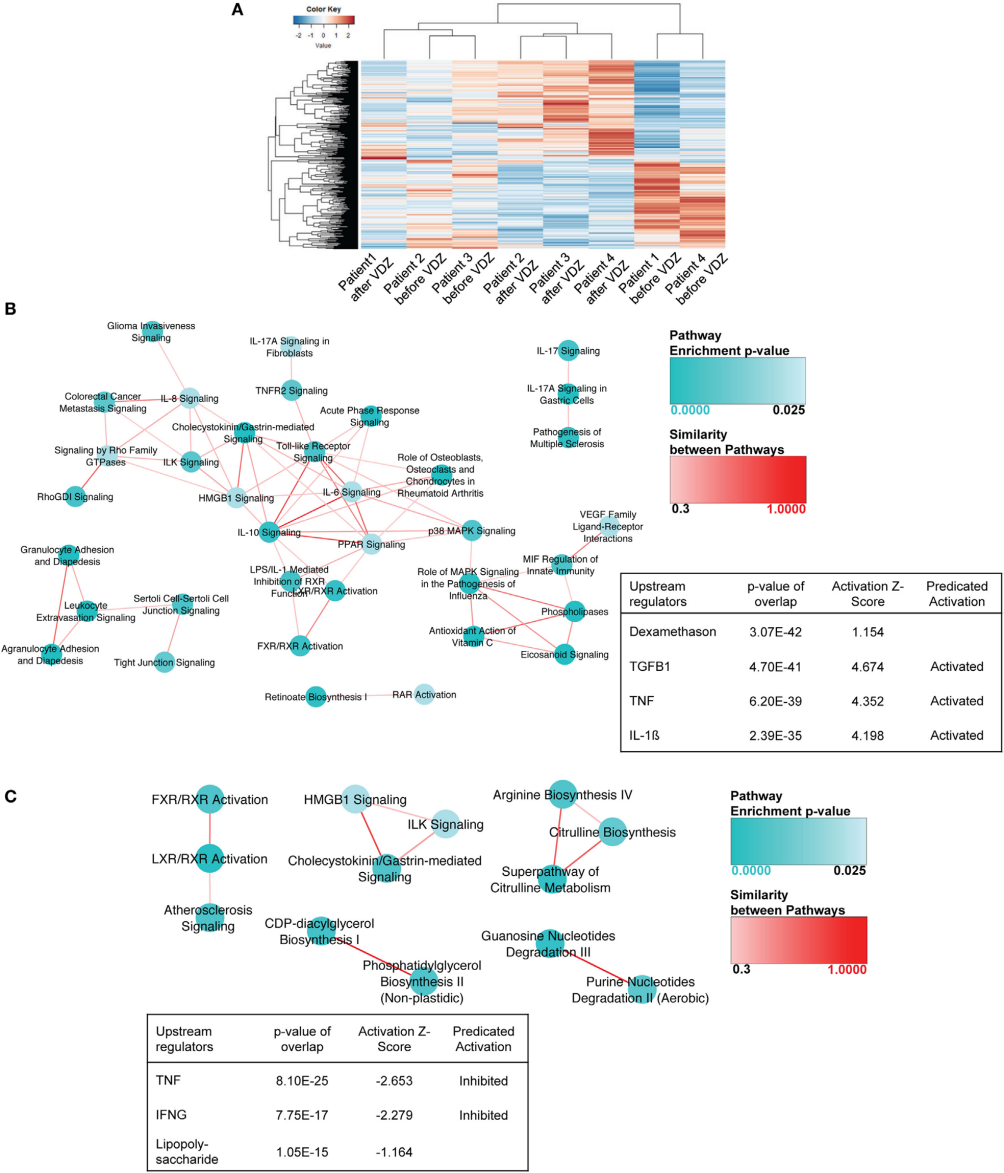
RNA sequencing in patients with ulcerative colitis (UC) under treatment with anti- α4β7 therapy with vedolizumab (VDZ). **(A)** Unsupervised hierarchical clustering of the expression profiles of differentially expressed genes upon VDZ therapy. **(B)** Canonical pathways and upstream regulators in UC patients with no remission to VDZ therapy. Ingenuity Pathway Analysis (IPA) of the differentially regulate genes in UC before and after VDZ therapy revealed granulocyte and agranulocyte adhesion and diapedesis to be among the three most significantly regulated pathways and TGFβ1 and TNF were among the three most significantly regulated upstream regulators with a predicted level of activation. **(C)** Canonical pathways and upstream regulators in UC patients with remission to VDZ therapy. In VDZ remitters, LXR/RXR activation and guanosine nucleotides degradation III were the most significantly regulated pathways on IPA and TNF and IFNγ as pro-inflammatory cytokines and upstream regulators were predicted to be inhibited in UC patients with remission to VDZ therapy. Turquoise dots represent pathway enrichment *p*-values; similarity of the pathways is represented by red lines.

When comparing expression profiles in UC patients before and after VDZ therapy, a total of 565 genes were differently expressed, with 297 genes showing an increased expression and 268 genes showing a decreased expression upon VZD therapy (Table S5 in Supplementary Material). Hierarchical clustering revealed more variability across patients than observed for CD (Figure 2A). As already observed for CD, IPA of the differentially regulated genes in UC upon VDZ therapy revealed agranulocyte adhesion and diapedesis to be significantly regulated (Table S6 in Supplementary Material).

When performing similar comparative analyses according to therapeutic response in UC patients with disease remission defined *via* clinical disease activity scoring (Mayo Clinic score of 2 or lower and no subscore higher than 1) (11), we found a total of 788 genes to be differentially regulated in mucosal biopsies from non-remitters obtained at week 14 of VDZ therapy compared with matched samples obtained prior to VDZ induction in the same patients. Baseline disease and clinical characteristics before and at week 14 of VDZ therapy are comparatively shown for UC patients with remission to VDZ induction therapy and those exhibiting no remission toward VDZ in Table 2. No significant confounder regarding therapeutic response could be found in this comparative listing.

**Table 2:** Clinical data of ulcerative colitis (UC) patients included for RNA sequencing.

	UC			
	Remitters (*n* = 2)		Non-remitters (*n* = 2)	
**Baseline characteristics**				

Sex (m/f)	2/0		2/0	
Age (years)				
Mean ± SD	27.9 ± 7.2		38.5 ± 22	
Disease manifestation				
Rectum and sigmoid colon only				
Left-sided				
Pancolitis	2		2	
Disease duration				
Mean ± SD (years)	4 ± 0		5.5 ± 2.1	
Prior anti-TNF treatment	2		2	
Concomittant medication				
Glucocorticoids only			2	
Immunosuppressants only^a^				
No glucocorticoids and immunosuppressants	2			

	**Before vedolizumab (VDZ)**	**VDZ week 14**	**Before VDZ**	**VDZ week 14**

Disease activity^b^				
Total Mayo Score, mean ± SD	7.5 ± 3.5	3 ± 1.4	6 ± 1.4	6.5 ± 2.1
Mayo Endoscopic Score, mean ± SD	2 ± 1.4	1 ± 1.4	5.6 ± 4.9	6.3 ± 5.5
Leukocyte count ± SD (×10^9^/L)	9.75 ± 2.1	10.3 ± 0.2	10.3 ± 1.9	10.8 ± 2.2
CrP ± SD (mg/L, reference < 5)	3.2 ± 3.2	8.2 ± 3.8	1.9 ± 0.4	3.9 ± 5.3

*^a^Immunosuppressants included azathioprine, methotrexate, and mercaptopurine*.

*^b^The partial Mayo Score ranges from 0 to 9, with higher scores indicating more active disease. The total Mayo Score consists of partial Mayo Score + Mayo Endoscopic Score and ranges from 0 to 12. Disease remission was defined as a Mayo Clinic score of 2 or lower and no subscore higher than 1*.

Importantly, as observed for CD, among those genes significantly upregulated by at least 1.5-fold in UC patients with no remission to VDZ therapy, many were involved in the recruitment of T cells and the T cell polarization such as the CXCR3 binding cytokines CXCL9, CXCL10, and CXCL11 (41–46), the Th2-dependent cytokine CCL26 (47), or the receptor of the Th2-dependent cytokine IL-13RA2 (48, 49) and the p40 containing cytokine IL23A, which has previously been shown to be essential for T cell-mediated colitis (39) (Table S7 in Supplementary Material). Furthermore, several members of the matrix metalloproteinase (MMP) family were upregulated such as MMP-1, MMP-3, MMP7, ADAM12, ADAMTS12, and ADAMTS2 consistent with the known role of MMPs for tissue degradation, the persistence of the inflammatory state, and fibrosis associated with IBD (50). When analyzing underlying signaling pathways in the differentially expressed genes in UC patients with no remission to therapy, both granulocyte adhesion and diapedesis and agranulocyte adhesion and diapedesis were among the three most significantly regulated pathways, and both IL1β and TNF were among the most significantly modulated upstream regulators, with a predicted activation of both upstream regulators on IPA (Figure 2B).

By contrast, as already observed for CD, the pro-inflammatory signature was considerably less present in UC patients with remission to VDZ therapy. In VDZ remitters, a total of 504 genes were differentially regulated before and at week 14 of VDZ therapy, and the only cyto- or chemokines with broad immunogenic properties that were upregulated by at least 1.5-fold were TNFSF9, IL-11, CXCL6, and IL-34 (Table S8 in Supplementary Material). Conversely, when performing IPA in these patients, the two most significantly modulated upstream regulators were TNF and IFNγ with a predicted level of inhibition instead of activation as observed in UC patients who showed no remission to VDZ treatment (Figure 2C).

Taken together, these data substantiate the concept that distinct gene signatures characterize remitters and non-remitters to VDZ treatment. In fact, IBD patients with no remission to VDZ therapy are characterized by a persistent activation of TNF-dependent signaling pathways and genes that mediate mucosal inflammation and tissue destruction while induction of remission with VDZ was associated with a decreased inflammatory signature and downregulated TNF signaling.

### α4β7 Expression on Lymphocyte Subsets in Patients With IBD

**Figure 3:**
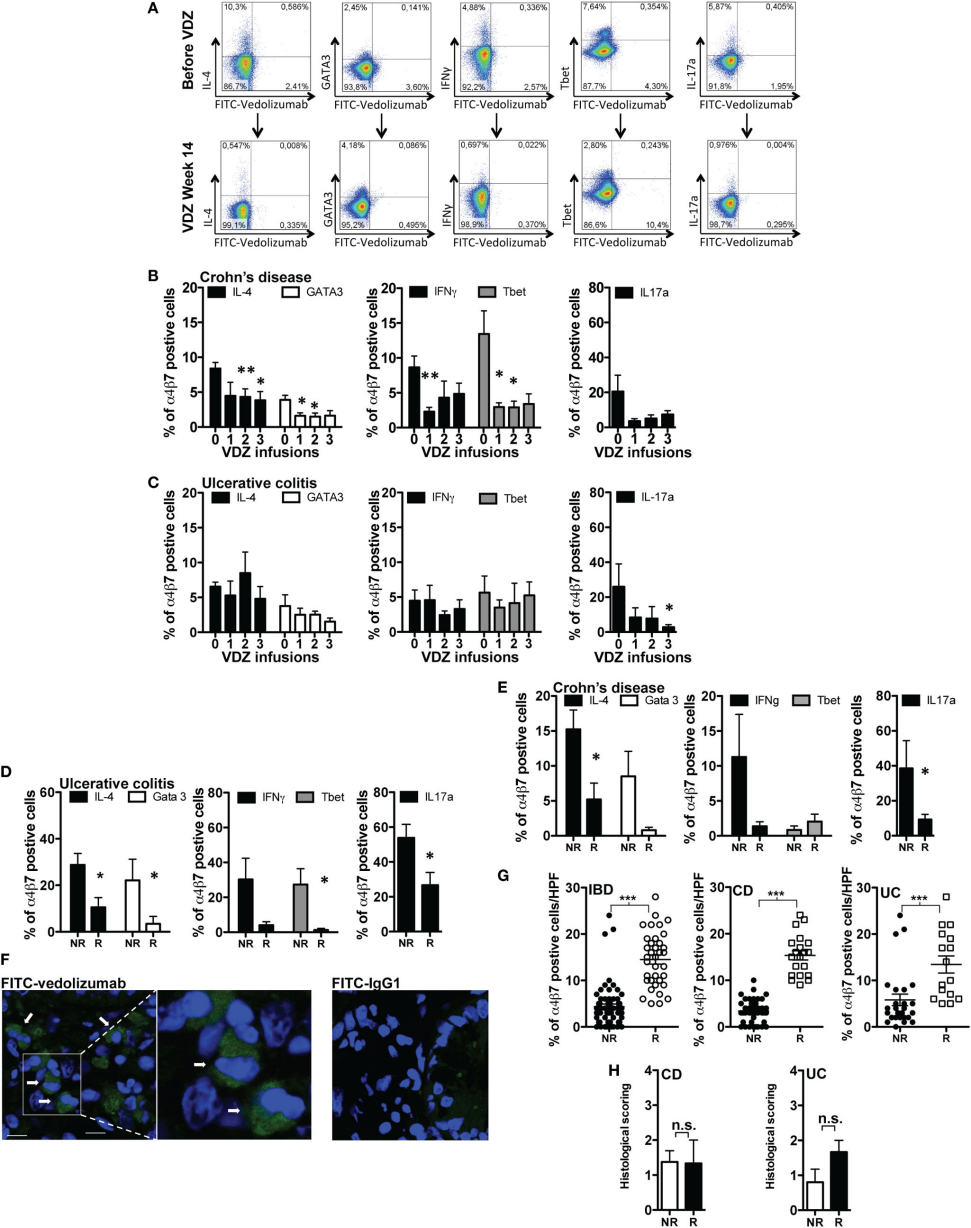
α4β7 expression on lymphocyte subsets in inflammatory bowel disease (IBD) patients. **(A)** Representative flow cytometric stainings of α4β7 expression on Th1, Th2, and Th17 polarized CD4^+^ T cells (previously gated on CD3 and CD4) before and after induction therapy with vedolizumab (VDZ) (week 14). Gray line: isotype control. **(B,C)** Quantification of α4β7 expression in different subsets of peripheral T cells (previously gated on CD3, CD4, and T-bet, IFNγ, IL-4, GATA-3, or IL-17, respectively) from patients with Crohn’s disease (CD) [**(B)**, *n* = 12] and ulcerative colitis (UC) [**(C)**, *n* = 10] before initiation of anti-integrin therapy with VDZ (indicated by “0”) (week 0) and directly before the second (week 2), third (week 6), and fourth (week 14) administration of 300 mg VDZ (indicated by “1,” “2,” and “3,” respectively). **(D,E)** Quantitative comparison of α4β7 expression in different subsets of lamina propria T cells in patients with UC [**(D)**, *n* = 11] and CD [**(E)**, *n* = 12]. α4β7 expression was compared between remitters and non-remitters for each disease after completed induction therapy with VDZ (UC: *n* = 5 remitters, *n* = 6 non-remitters; CD: *n* = 6 remitters, *n* = 6 non-remitters). **(F)** Cryosections of colonic biopsies from IBD patients were stained with fluorescein isothiocyanate (FITC)-VDZ or FITC-IgG1. After counterstaining with DAPI (Thermo Fischer Scientific, Cat. D1306), cryosections were analyzed with confocal microscopy. The arrows indicate α4β7-expressing mucosal cells. **(G)** The amount of α4β7-expressing cells within the lamina propria was assessed in 15 IBD patients (8 CD and 7 UC) patients prior to the commencement of VDZ therapy. For each patient a mean of 5 high power field (HPF) were analyzed by fluorescence microscopy and the amount of α4β7-expressing cells prior to VDZ therapy was quantified. UC and CD patients with clinical remission toward subsequent VDZ exhibited significantly higher levels of α4β7-expressing cells within the lamina propria compared with non-remitters. **(H)** Histopathological scoring from the same remitters and non-remitters [as shown in **(G)**] prior to VDZ therapy. Results are presented as mean ± SEM. Differences between samples are compared with the Mann–Whitney *U* test (*p < 0.05; **p < 0.01; ***p < 0.001).

Based on the above findings, we hypothesized that systemic and mucosal leukocytes within the lamina propria of the gut might also show certain immunologic properties that define or indicate therapeutic response to VDZ therapy. In a first set of experiments, we thus quantified α4β7 expression on different subsets of T cells in IBD patients before as well as after induction therapy with VDZ. For this purpose, we labeled commercially available VDZ and a human IgG1 isotype with FITC. Staining with FITC-VDZ allowed a clear discrimination of α4β7 expression on peripheral blood CD3^+^, CD4^+^, and CD8^+^ positive T cells, on B cells (CD19^+^), macrophages (CD14^+^), and on natural killer (NK) cells (CD56^+^) (Figure S1A in Supplementary Material) as well as on Th1, Th2, and Th17 polarized CD4^+^ T cells, as defined by the expression of IFNγ or T-bet and IL-4 or IL-13 or GATA3 and IL-17a, respectively (Figure 3A). Prior to VDZ therapy, highest expression of α4β7 was observed in B cells (34%), followed by CD8^+^ T cells (27%), CD3^+^ T cells (18%), and CD4^+^ T cells (20%) (Figure S1B in Supplementary Material) from IBD patients. Importantly, two applications of 300 mg VDZ led to a significant decline of α4β7 expression on peripheral CD3^+^, CD4^+^, and CD8^+^ T cells as well as in B cells and NK cells (Figure S1C in Supplementary Material). Consistent with the robust decline of α4β7 expression on overall peripheral CD4^+^ T cells, anti-α4β7 therapy with VDZ led to a progressive decline of α4β7 expression in Th1, Th2, and Th17 polarized CD4^+^ T cells from patients with CD (Figure 3B) and in Th17 polarized CD4^+^ T cells from UC patients under anti-α4β7 therapy with VDZ (Figure 3C).

We then turned our attention to α4β7 expression on gut immune cells of IBD patients. Of note, when quantifying α4β7 expression in LPMCs from seven IBD patients prior to initiation of VDZ therapy, we observed similar expression levels compared with those seen in peripheral blood lymphocytes with highest expression in mucosal CD19^+^ B cells (32%) and CD3^+^ (20%), CD4^+^ (11%), and CD8^+^ (25%) T cells (Figure S1D in Supplementary Material). To address the potential relevance of altered intestinal α4β7 expression for driving and perpetuating mucosal inflammation in IBD, we comparatively analyzed differences of α4β7 expression in lamina propria T cell subsets in remitters and non-remitters to VDZ treatment (11 UC, 5 remitters, 6 non-remitters; 12 CD, 6 remitters, 6 non-remitters), with disease remission defined *via* clinical disease activity scoring [UC: Mayo Clinic score of 2 or lower and no subscore higher than 1 (11); CD: HBI < 5 (19)]. Importantly, in UC patients with clinical remission upon anti-integrin therapy with VDZ, we observed a significantly decreased α4β7 expression in Th1 polarized lamina propria CD4^+^ T cells, as defined by expression of IFNγ or the transcription factor T-bet, in Th2 polarized lamina propria CD4^+^ T cells expressing either IL-4 or the transcription factor GATA3, as well as in Th17 cells expressing IL-17a (Figure 3D). In CD patients, a significant reduction of α4β7 expression on Th2 and Th17 lamina propria CD4^+^ T cells was likewise observed in VDZ remitters compared with CD non-remitters, whereas the reduction of α4β7 expression on Th1 polarized CD4^+^ T cells did not reach statistical significance (Figure 3E).

To further substantiate these findings, we hypothesized that the amount of α4β7-expressing mucosal cells prior to the initiation of VDZ therapy might be directly related to the outcome and therapeutic efficacy of subsequent anti-α4β7 VDZ therapy. To address this question, we first verified that FITC-VDZ allowed immunohistochemical identification of α4β7-expressing cells in the lamina propria in intestinal cryosections of IBD patients compared with staining with a FITC-IgG1 isotpye control (Figure 3F). To analyze whether the initial amount of α4β7-expressing cells in the lamina propria of IBD patients correlated to the outcome of anti-α4β7 therapy with VDZ, we assessed α4β7 expression in gut immune cells in cryosections from 15 IBD patients (8 CD and 7 UC) prior to the commencement of VDZ therapy. Of these 15 IBD patients, 7 patients (3 UC and 4 CD) exhibited remission at week 14 of VDZ therapy as defined by clinical scoring. Importantly, these remitters were characterized by significantly higher levels of α4β7 expression on mucosal immune cells prior to VDZ therapy initiation compared with non-remitters (Figure 3G). As shown in Figure 3G, in the cohort of IBD patients, remitters were characterized by a mean number of 14.5 α4β7^+^ cells per HPF in the lamina propria prior to the initiation of VDZ therapy compared with 4.4 α4β7-expressing cells per HPF in patients which showed no remission to subsequent VDZ therapy (*p* < 0.0001). Similar observations were made when UC and CD patients were analyzed separately. CD patients with remission upon VDZ were characterized by a mean number of 15.4 α4β7^+^ cells/HPF prior to commencement of VDZ therapy compared with only 3.4 α4β7-expressing cells/HPF in non-remitters (*p* < 0.0001). Similarly, UC patients with remission to subsequent VDZ therapy exhibited significantly higher amounts of α4β7^+^ cells in the lamina propria before VDZ therapy compared with non-remitters (13.4 vs. 5.8, *p* = 0.0003). Importantly, for both CD and UC, there were no differences in histopathologic scoring prior to VDZ therapy between remitters and those without remission toward subsequent anti-integrin therapy with VDZ (Figure 3H). This observation excludes differences in histological baseline disease activity in remitters and non-remitters before initiation of VDZ therapy as a potential confounder of therapeutic efficacy and thus confirms that the amount of α4β7^+^ cells within the lamina propria might represent a determinant for subsequent outcome of anti-integrin with VDZ. Furthermore, UC and CD patients with remission toward VDZ did not exhibit differences in leukocyte counts, serum CrP levels, clinical disease activity, concomitant use of glucocorticosteroids and immunosuppressant, or prior exposure to anti-TNF therapy compared with non-remitters (Table 3), thereby corroborating that remitters and non-remitters to VDZ exhibited similar clinical parameters of disease activity and severity as well. Individual phenotypical and clinical characteristics of every patient included in each experiment are shown in Table S9 in Supplementary Material.

**Table 3:** Clinical data of inflammatory bowel disease patients with α4β7 quantification prior to anti-integrin therapy with vedolizumab.

	Ulcerative colitis (UC)		Crohn’s disease (CD)	
	Remitter (*n* = 3)	Non-Remitter (*n* = 4)	Remitter (*n* = 4)	Non-Remitter (*n* = 4)
**Baseline characteristics**				

Sex (m/f)	2/1	3/1	1/3	1/3
Age (years)				
Mean ± SD	46.6 ± 12.9	35.6 ± 13.6	38.9 ± 15.8	27.9 ± 7.4
Leukocyte count ± SD (×10^9^/L)	10 ± 2.1	9.8 ± 1.6	9 ± 4.9	11.1 ± 6.2
CrP ± SD (mg/L, reference < 5)	10 ± 2.1	9.8 ± 1.6	9.3 ± 7.6	18.1 ± 29

**Disease characteristics**				

Extend, UC				
Rectum and sigmoid colon only	1	1		
Left-sided				
Pancolitis		1		
Extend, CD	2	2		
Ileum				
Ileum + colon			2	3
Ileum + colon + upper GI tract			2	1
Disease duration				
Mean ± SD (years)	8.3 ± 3.2	6.75 ± 5.3	9 ± 4.9	11.3 ± 8
Disease activity, UC^a^				
Partial Mayo Score, mean ± SD	5.6 ± 2.1	6 ± 2.2		
Mayo Endoscopic Score, mean ± SD	2.3 ± 1.2	2 ± 0.8		
Disease activity, CD				
HBI, mean ± SD^b^			13 ± 2.6	13.2 ± 7.8
SES-CD, mean ± SD^c^			13.6 ± 6.7	16.3 ± 2.5
Prior anti-TNF treatment	3	3	4	4
Concomittant medication				
Glucocorticoids only	1	1	1	4
Immunosuppressants only^d^			1	
No glucocorticoids and immunosuppressants	2	3	2	
Prednisone-equivalent dose (mg)				
Mean ± SD	7.5 ± 0	20 ± 0	20 ± 0	22.5 ± 13.4

*^a^The partial Mayo Score ranges from 0 to 9, with higher scores indicating more active disease. The total Mayo Score consists of partial Mayo Score + Mayo Endoscopic Score and ranges from 0 to 12. Disease remission was defined as a Mayo Clinic score of 2 or lower and no subscore higher than 1*.

*^b^The Harvey–Bradshaw index (HBI) ranges from 0 to 26, with higher scores indicating more active disease. Disease remission was defined as a HBI < 5*.

*^c^The Simple Endoscopic Score for Crohn’s Disease (SES-CD) assesses ulcer size, ulcerated surface, surface affected from disease and the presence of stenosis and ranges from 0 to 56, with higher scores indicating more severe disease*.

*^d^Immunosuppressants included azathioprine, methotrexate, and mercaptopurine*.

Taken together, our findings suggest that the amount of mucosal α4β7-expressing cells prior to the initiation of VDZ therapy might well function as an *a priori* indicator or predictive biomarker for therapeutic efficacy of subsequent VDZ therapy. Hence, the quantification of α4β7 expression within the lamina propria might allow for stratification of IBD patients according to their individual expression of the therapeutic target and the associated likelihood to achieve subsequent therapeutic remission to the VDZ treatment directed against it.

### Cellular Modes of Action of VDZ

**Figure 4:**
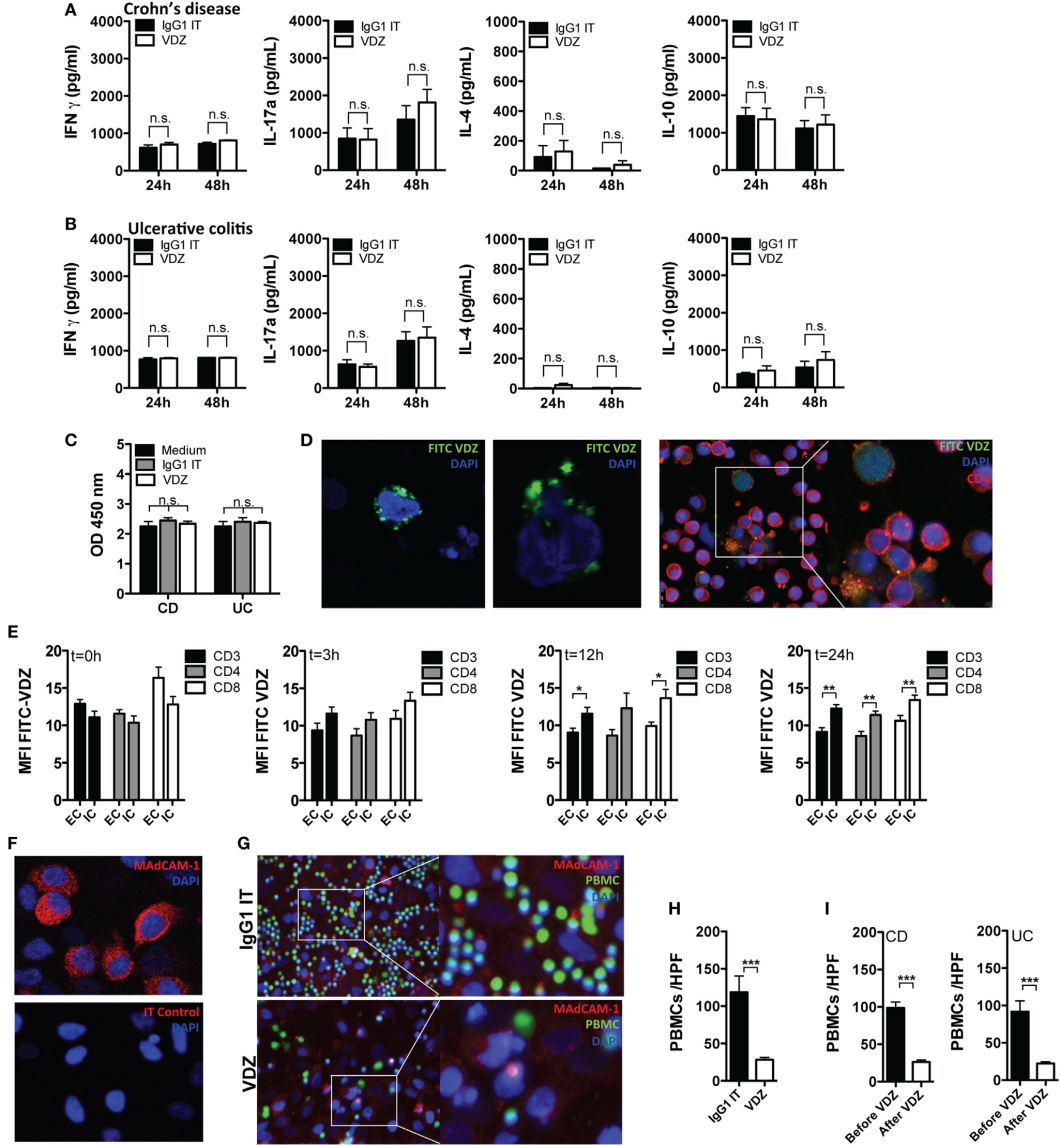
Cellular mechanism of action of vedolizumab (VDZ). **(A,B)** Peripheral blood leukocytes from patients with Crohn’s disease (CD) [**(A)**, *n* = 7] and ulcerative colitis (UC) [**(B)**, *n* = 7] were incubated with VDZ or isotype (IT) control for 24 and 48 h. Afterward, concentration of IFNγ, IL-4, IL17a, and IL-10 in the supernatant was quantified by ELISA. **(C)** Peripheral blood leukocytes from patients with CD (*n* = 7) and UC (*n* = 7) were incubated with VDZ, IT, or media only for 24 h. Afterward, release of cytoplasmic histone-associated DNA fragments as a marker of induced cell death was quantified by ELISA. **(D)** Confocal microscopy of peripheral blood leukocytes (left panels) and sorted CD4^+^ T cells (right panels) incubated for 24 h with fluorescein isothiocyanate (FITC)-labeled VDZ. **(E)** Internalization of α4β7 after binding to VDZ over time. Peripheral leukocytes from seven inflammatory bowel disease patients were incubated for the indicated time points with VDZ. From each patient, 5 × 10^5^ peripheral leukocytes were first permeabilized to make the intracellular compartment accessible for staining followed by staining with fluorescent labeled VDZ. Another 5 × 10^5^ cells were first stained with FITC-VDZ and were then permeabilized. Afterward, α4β7 expression was quantified in CD3^+^, CD4^+^, and CD8^+^ T cells. **(F)** Mucosal addressin cell adhesion molecule-1 (MAdCAM-1) expression in HeLa cells. Upper panel: staining of MAdCAM-1. Lower panel: isotype control. **(G,H)** Peripheral blood leukocytes from healthy controls (*n* = 4) were labeled with a vital dye (green) and co-incubated with IgG1 and VDZ for 60 min on confluent grown MAdCAM-1 expressing HeLa cells (red). Afterward, non-attaching cells were removed by repeated washing. Adhering cells were visualized with fluorescence microscopy and attaching leukocytes per high power field (HPF) were counted. **(I)** Peripheral leukocytes from CD (*n* = 3) and UC (*n* = 3) patients before and after completion of VDZ induction therapy from the same patient were labeled with a vital dye and co-incubated with IgG1 and VDZ for 60 min on confluent grown MAdCAM-1 expressing HeLa cells. Afterward, non-attaching cells were removed by repeated washing. Adhering cells were visualized with fluorescence microscopy and attaching leukocytes per HPF were counted. Results are presented as mean ± SEM. Differences between samples are compared with the Mann–Whitney *U* test (*p < 0.05; **p < 0.01; ***p < 0.001).

Having shown that the adhesion and diapedesis of granulocytes and agranulocytes is among the most significantly regulated pathways under VDZ therapy and that α4β7 expression shows a progressive decline on effector T cells upon VDZ therapy, we hypothesized that surface α4β7 is internalized under VDZ therapy, leading to an impaired adhesion of lymphocytes. At the same time, we aimed to rule out other major cellular and immunological modes of action of VDZ. Since the induction of cytokine release by therapeutic antibodies is a known side effect that can have deleterious clinical consequences (51), we first analyzed whether VDZ alters or induces the expression of various cytokines. For this purpose, peripheral blood leukocytes from IBD patients were incubated with VDZ or IgG1 Isotype control for 24 and 48 h. Based on pharmacokinetic data that IBD patients responding to VDZ therapy exhibit therapeutic serum trough levels from 11 to 38 μg/mL (11, 12), we chose a concentration of 40 µg/mL to study *in vitro* effects of VDZ. Incubation of peripheral leukocytes with VDZ did not elicit release of the Th1 cytokine IFNγ, the Th2 cytokine IL-4, the Th17 cytokine IL17a, or IL-10 in cultivated leukocytes from patients with CD (Figure 4A) or UC (Figure 4B) compared with isotype control treated cells. To rule out induction of programmed cell death by VDZ, we then followed a similar approach and measured the release of cytoplasmic histone-associated DNA fragments as an established marker of induced cell death. Importantly, there was no difference in the quantity of histone-associated DNA fragments in leukocytes that have been incubated with VDZ, isotype control, or media only (Figure 4C).

To gain first insights into the potential internalization of α4β7 after binding to VDZ, we co-incubated peripheral blood leukocytes with fluorescent labeled VDZ and visualized cells with confocal microscopy. As shown in Figure 4D, after 24 h of incubation at 37°C, we observed a punctate perinuclear staining pattern indicative of a successfully cellular internalized α4β7 receptor complex. Furthermore, a clear intracellular FITC signal was present in sorted peripheral CD4^+^ T cells from IBD patients after 24 h of incubation with FITC-VDZ (Figure 4D). Based on these observations, we then quantified internalization of α4β7 after binding to VDZ in peripheral leukocytes from seven IBD patients over a specified time period in flow cytometric analyses (Figure 4E). After incubation of peripheral blood leukocytes for different periods of time with VDZ, half of the cells from each patient were first permeabilized to make the intracellular compartment accessible for staining, followed by staining with fluorescent labeled VDZ (upstream permeabilization) while the other half of the cells were first stained with FITC-VDZ and then permeabilized (downstream permeabilization). As shown in Figure 4E, there was no difference in the FITC MFI between upstream and downstream permeabilized cells at baseline. However, over time we observed a progressive increase in the FITC MFI in CD3^+^, CD4^+^, and CD8^+^ T cells, in which the intracellular compartment has been made accessible prior to staining with FITC-VDZ and upstream permeabilized cells exhibited a significantly higher FITC MFI after 12 and 24 h compared with downstream permeabilized cells. These results therefore indicate that after addition of VDZ, surface α4β7 is internalized over time with a peak of the receptor internalization after 12 and 24 h.

To demonstrate that α4β7 internalization by VDZ directly impairs the interaction with MAdCAM-1, we established *in vitro* adhesion assays using MAdCAM-1 expressing HeLa cells. First, strong MAdCAM-1 expression in HeLa cells was verified by immunohistochemistry (Figure 4F). HeLa cells were then grown to confluence and after 2 days, peripheral leukocytes from healthy donors were labeled with a vital dye and preincubated with VDZ or IgG1 for 60 min. Subsequently, leukocytes were co-incubated with HeLa cells, and after washing away non-attaching cells, fixed cells were analyzed with fluorescence microscopy (Figure 4G). Importantly, leukocytes preincubated with VDZ were significantly impaired to adhere to MAdCAM-1 expressing HeLa cells when compared with IgG1 treated cells (Figure 4H).

In a similar set of experiments, adherence of peripheral leukocytes from IBD patients before and after completion of VDZ induction therapy was assessed. For this purpose, peripheral leukocytes from CD (*n* = 3) and UC (*n* = 3) patients were obtained and for each patient, leukocytes before and after completion of VDZ induction therapy were available, thereby directly allowing assessment of VDZ treatment on the adherence of leukocytes for each individual patient. As shown in Figure 4I, after washing away non-attaching cells, leukocytes derived from CD and UC patients after completed VDZ induction therapy were significantly impaired to adhere to MAdCAM-1 expressing HeLa cells when compared with matched leukocytes derived from the same patients prior to the initiation of VDZ therapy.

In their totality, these data provide evidence that the main mode of action by which VDZ acts on the cellular levels is indeed the internalization of surface α4β7, leading to the biological effect of impaired interaction with MAdCAM-1.

## Discussion

Vedolizumab is the first anti-integrin antibody therapy that has been approved for the treatment of UC and CD patients. Despite clinically proven therapeutic efficacy in up to 50% of treated IBD patients showing clinical response upon VDZ induction therapy and a favorable safety profile, data from large clinical studies show that VDZ is not effective in subgroups of IBD patients. Given these considerations, it is important not only to understand the biologic mode of action but also to characterize differences between responders and non-responders to anti-integrin therapy with VDZ on the cellular and molecular level. In this study, we therefore set off to explore factors that define clinical response toward VDZ and to delineate the immunologic differences between responders and non-responders to anti-integrin therapy with VDZ.

In an initial approach, we performed whole transcriptome analysis using RNA Sequencing in UC and CD patients responding to VDZ compared with non-responders. For both diseases, we found that failure of VDZ to induce clinical remission was associated with an upregulation of several pro-inflammatory and immunogenic genes such as several pro-inflammatory chemo- and cytokines or its receptors and a tissue destructive signature as indicated by the upregulation of a large panel of genes encoding for various proteases. Furthermore, IPA revealed a strong activation of TNF-dependent signaling in non-remitters to VDZ treatment. By contrast, in UC and CD patients with remission to VDZ treatment, no clear pro-inflammatory or immunogenic signature was present. Instead, in UC patients with remission to VDZ, TNF, and IFNγ as broad and potent pro-inflammatory cytokines were predicted to be inhibited instead of activated on Ingenuity analyses.

Importantly, endoscopic and clinical parameters of disease severity such as leukocyte counts, serum CrP levels, clinical disease activity, concomitant use of glucocorticosteroids and immunosuppressant, or prior exposure to anti-TNF therapy were not different between remitters and non-remitters before initiation of VDZ therapy. Thus, it can be ruled out that the observed regulations of the above-mentioned genes and related pathways are due to pre-existent differences in disease severity between remitters and non-remitters but can be rather related to VDZ treatment itself.

To further delineate the effects of VDZ treatment on the expression of α4β7 on systemic and gut immune cells, we comparatively quantified the amount of surface α4β7 on various systemic and intestinal effector T cells. Interestingly, α4β7 expression showed a progressive decline on systemic Th1, Th2, and Th17 polarized CD4^+^ T cells from patients with CD and on Th17 cells from patients with UC. These observations fit well to our mechanistic studies on the mode of action of VDZ therapy. Using flow cytometric analyses and confocal microscopy, we were able to show that treatment with VDZ leads to internalization of the α4β7 receptor which results in significantly impaired interaction with and adherence to MAdCAM-1 as shown *in vitro* in this report, while cellular effects such as cytokine production or the induction of programmed cell death remained unaltered. These data are consistent with observations made in clinical trials in which no changes in serum levels of cytokines such as TNF, IL-1, or IFNγ were observed under VDZ treatment (10–12, 52, 53). Furthermore, during the antibody engineering of VDZ, point mutations were made to the Fc receptor (FcR) binding motif ELLGGP. As such, Leu239 and Gly241 were mutated to Ala to reduce FcR binding, and the unaltered cytokine production and apoptosis induction very well fit to the lack of elicitation of FcR effector functions from VDZ and results of a previous study which has shown that VDZ does not induce complement-dependent cytotoxicity and antibody-dependent cytotoxicity (54). Consistent with the internalization observed *in vitro* and the progressive decline of surface α4β7 expression during VDZ induction therapy, we were able to show in functional studies that cells exposed to exogenously added VDZ as well as cells taken from patients after completed VDZ induction therapy are significantly impaired in their attachment to MAdCAM-1 expressing cells. Hence, the observed progressive decrease of surface α4β7 on peripheral effector T cells during VDZ induction therapy might well reflect VDZ-induced internalization of α4β7. One might argue that receptor internalization and re-surfacing of unbound and biologically active receptor is a continuous process, leading to the re-appearance of α4β7 on the surface. However, in this regard, it is important to note that the mean half-life of VDZ is approximately 15–22 days and it has been shown in clinical trials that, after administration of VDZ at weeks 0 and 2, followed by dosing at every fourth or eighth week, greater than 95% of α4β7 receptors were saturated (11, 12). Hence, it appears plausible that steady-state serum concentrations of VDZ lead to immediate binding and subsequent internalization of re-surfacing or newly synthesized α4β7 receptor, so that the majority of α4β7 is internalized at steady-state serum levels of VDZ.

Based on the decline of α4β7 on the surface of effector T cells during VDZ therapy, we expected that also a decrease of α4β7-expressing cells within the lamina propria of IBD patients would be noted after completed VDZ induction therapy. Indeed, both UC and CD patients with remission to VDZ therapy exhibited a robust decrease of α4β7 on Th1, Th2, and Th17 polarized CD4^+^ T cells after completed VDZ induction therapy compared with non-remitters.

To allow a timely, targeted, and economic treatment regimen with biological agents, the identification of factors that predict therapeutic responses to treatment is of central importance for the management of IBD patients. Recently, our group demonstrated that the amount of mTNF-expressing cells in the lamina propria of CD patients prior to the initiation of anti-TNF therapy is directly related to the outcome of subsequent anti-TNF therapy (55). Specifically, patients with high numbers of mTNF^+^ cells showed significantly higher short-term response rates at week 12 upon subsequent anti-TNF treatment when compared with patients with low amounts of mTNF^+^ cells (55). Furthermore, the clinical response in patients with high mTNF^+^ cells was sustained over a follow-up period and was associated with mucosal healing (55). In addition, exploratory studies from an UC clinical phase II trial with the anti-adhesion molecule antibody etrolizumab, which binds to the β7 subunit of the α4β7 and αEβ7 integrins, strengthen the approach of assessing the expression of the target molecule of anti-adhesion molecule antibody treatment. In this regard, it was shown *via* immunofluorescence staining that high expression of αE-positive cells in colonic biopsies at baseline was directly related to the therapeutic response upon etrolizumab treatment (56).

In this report, we followed a similar approach and quantified α4β7 expression in colonic biopsies from UC and CD patients before the commencement of anti-integrin therapy with VDZ. Importantly, UC and CD patients with remission to subsequent VDZ therapy exhibited significantly higher amounts of α4β7-expressing cells at baseline compared with non-remitters. These data further substantiate the concept that the expression of the target molecule can be used as a prognostic marker for therapeutic response, and to the best of our knowledge, our report is the first to demonstrate that the expression of α4β7 at baseline might well be used for prediction of therapeutic responses toward subsequent anti-integrin therapy with VDZ.

Limitations of the study should also be discussed, one of which is the low number of patients included in RNA sequencing, thereby rendering these results exploratory in nature. However, our approach of comparatively including both UC and CD remitters and non-remitters before and at week 14 of VDZ therapy allowed to analyze drug effects and difference between remitters and non-remitters while controlling for patient-specific factors. Furthermore, we used colonic biopsies for RNA sequencing so that changes in the transcriptome cannot be related to a certain cell type. Given these considerations, it seems clears that results should be corroborated in larger patient cohort using more specific sequencing analyses such as single-cell sequencing.

In summary, we have shown that remitters and non-remitters to VDZ therapy exhibit distinct genetic signatures, which are characterized by the upregulation of genes that mediate mucosal inflammation and tissue destruction in IBD patients with remission to VDZ treatment. Furthermore, RNA sequencing revealed a strong activation of TNF-dependent signaling pathways in non-remitters to VDZ treatment, thereby providing first evidence that anti-TNF therapy might be therapeutically effective in IBD patients with VDZ failure. Treatment with VDZ was associated with a progressive decline of α4β7 expression on the surface of peripheral leukocytes while therapeutic remission was characterized by significantly higher amounts of α4β7-expressing cells in the lamina propria of CD and UC patients at baseline compared with non-responders. Hence, quantification of α4β7-expressing cells within the lamina propria might represent a novel approach which may allow prediction of therapeutic success prior to the initiation of anti-integrin therapy with VDZ, thereby opening up new avenues for personalized medicine in IBD patients.

## Availability of Data

All data generated or analyzed during this study are included in this published article (and its supplementary files). RNA sequencing data have been submitted to the Sequence Read Archive (study accession number SRP151738).

## Ethics Statement

The study was carried out in accordance with the recommendations of the ethical committee of the University Hospital, Friedrich-Alexander-Universität Erlangen-Nürnberg, Germany. The protocol was approved by the ethical committee of the University Hospital, Friedrich-Alexander-Universität Erlangen-Nürnberg, Germany. Each patient gave written informed consent in accordance with the Declaration of Helsinki before inclusion into the study.

## Author Contributions

All the authors made substantial contributions to the conception and design of the study (or the acquisition of data, or analysis and interpretation of data), the drafting the article (or critically revising it for important intellectual content), and to the final approval of the version to be submitted. TR performed the experiments and wrote the manuscript and together with MN and RA designed the study. UB performed experiments, helped in study design, and critically revised the manuscript for important intellectual content. FF and AE performed RNA sequencing and analyzed sequencing data. MV performed histopathological scoring and critically revised the manuscript. MN and RA designed the study and critically revised the manuscript.

## Conflict of Interest Statement

The authors declare that the research was conducted in the absence of any commercial or financial relationships that could be construed as a potential conflict of interest.

## Supplementary Material

Figure S1 α4β7 expression on lymphocyte subsets in inflammatory bowel disease (IBD) patients. **(A)** Staining of α4β7 expression on CD3^+^, CD4^+^, and CD8^+^ positive T cells, on B-cells (CD19^+^), macrophages (CD14^+^), and on natural killer (NK) cells (CD56^+^) with fluorescein isothiocyanate (FITC)-labeled vedolizumab (VDZ) on lamina propria lymphocytes. Gray line: isotype control. **(B)** Quantification of α4β7 expression by flow cytometry in different subsets of peripheral leukocytes from 11 IBD patients prior to VDZ therapy. **(C)** α4β7 expression in peripheral blood lymphocytes in 13 IBD patients before VDZ therapy (indicated by “0”) (week 0) and directly before the second (week 2) and third (week 6) administration of 300 mg VDZ (indicated by “1” and “2,” respectively). **(D)** Quantification of α4β7 expression by flow cytometry in different subsets of lamina propria lymphocytes from five IBD patients. Results are presented as mean ± SEM. Differences between samples are compared with the Mann–Whitney *U* test.

Table S1 CD Genes before and after VDZ.

Table S2 CD pathways before and after VDZ.

Table S3 CD Non-Remitter Genes before and after VDZ.

Table S4 CD Remitter Genes before and after VDZ.

Table S5 UC Genes before and after VDZ.

Table S6 UC pathways before and after VDZ.

Table S7 UC Non-Remitter Genes before and after VDZ.

Table S8 UC Remitter Genes before and after VDZ.

Table S9 Individual phenotypical and clinical characteristics of patients included in each experiment.
