# Supplementary figures and images for: Effects of Anti-Integrin Treatment With Vedolizumab on Immune Pathways and Cytokines in Inflammatory Bowel Diseases
Source: Front Immunol. 2018 Jul 31;9:1700. doi: 10.3389/fimmu.2018.01700 (PMC6090141; doi:10.3389/fimmu.2018.01700)

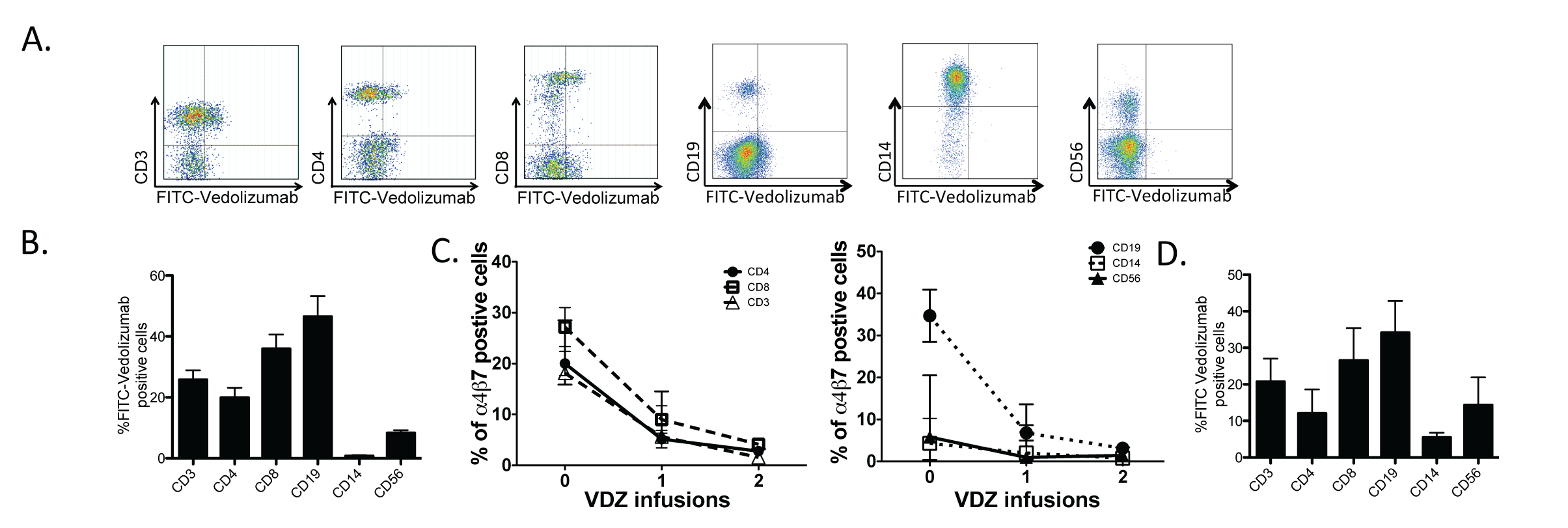

Supplement: Figure S1 — α4β7 expression on lymphocyte subsets in inflammatory bowel disease (IBD) patients. (A) Staining of α4β7 expression on CD3+, CD4+, and CD8+ positive T cells, on B-cells (CD19+), macrophages (CD14+), and on natural killer (NK) cells (CD56+) with fluorescein isothiocyanate (FITC)-labeled vedolizumab (VDZ) on lamina propria lymphocytes. Gray line: isotype control. (B) Quantification of α4β7 expression by flow cytometry in different subsets of peripheral leukocytes from 11 IBD patients prior to VDZ therapy. (C) α4β7 expression in peripheral blood lymphocytes in 13 IBD patients before VDZ therapy (indicated by “0”) (week 0) and directly before the second (week 2) and third (week 6) administration of 300 mg VDZ (indicated by “1” and “2,” respectively). (D) Quantification of α4β7 expression by flow cytometry in different subsets of lamina propria lymphocytes from five IBD patients. Results are presented as mean ± SEM. Differences between samples are compared with the Mann–Whitney U test. [file Image_1.tif]
