# Supplementary material for: Effects of Anti-Integrin Treatment With Vedolizumab on Immune Pathways and Cytokines in Inflammatory Bowel Diseases
Source: Front Immunol. 2018 Jul 31;9:1700. doi: 10.3389/fimmu.2018.01700 (PMC6090141; doi:10.3389/fimmu.2018.01700)
Supplement: Table S9 — Individual phenotypical and clinical characteristics of patients included in each experiment. [file Table_9.docx]

**Supplementary Table for Figure 1**

| **ID No.** | **Age** | **Gender** | **Disease** | **Montreal** |
| --- | --- | --- | --- | --- |
| 1 | 42 | f | CD | A2L3B1 |
| 2 | 51 | m | CD | A2L1B2B3p |
| 3 | 50 | m | CD | A2L3+L4B1 |
| 4 | 25 | f | CD | A1L3+L4B1 |
| 5 | 37 | f | CD | A2L1+L4B3p |
| 6 | 63 | f | CD | A3L3B1 |

**Supplementary Table for Figure 2**

| **ID No.** | **Age** | **Gender** | **Disease** | **Montreal** |
| --- | --- | --- | --- | --- |
| 7 | 57 | m | UC | E3 |
| 8 | 26 | m | UC | E3 |
| 9 | 36 | m | UC | E3 |
| 10 | 26 | m | UC | E3 |

S**upplementary Table for Figure 3b,c - PBMC**

| **ID No.** | **Age** | **Gender** | **Disease** | **Montreal** |
| --- | --- | --- | --- | --- |
| 2 | 51 | m | CD | A2L1B2B3p |
| 3 | 50 | m | CD | A2L3+L4B1 |
| 4 | 25 | f | CD | A1L3+L4B1 |
| 5 | 37 | f | CD | A2L1+L4B3p |
| 6 | 63 | f | CD | A3L3B1 |
| 7 | 57 | m | UC | E3 |
| 8 | 26 | m | UC | E3 |
| 9 | 36 | m | UC | E2 |
| 10 | 26 | m | UC | E3 |
| 11 | 40 | f | CD | A1L1B2 |
| 12 | 31 | f | CD | A2L3+L4B1 |
| 13 | 22 | m | CD | A1L3+L4B2 |
| 14 | 55 | m | CD | A2L1B2 |
| 15 | 52 | f | CD | A2L3+L4B3p |
| 16 | 39 | f | CD | A1L1B2B3p |
| 17 | 26 | m | CD | A2L3+L4B3p |
| 18 | 55 | m | UC | E3 |
| 19 | 51 | m | UC | E3 |
| 20 | 58 | f | UC | E2 |
| 21 | 66 | m | UC | E2 |
| 22 | 55 | m | UC | E3 |
| 23 | 30 | m | UC | E3 |

**Supplementary Table for Figure 3d,e - LPMC**

| **ID No.** | **Age** | **Gender** | **Disease** | **Montreal** |
| --- | --- | --- | --- | --- |
| 1 | 42 | f | CD | A2L3B1 |
| 2 | 51 | m | CD | A2L1B2B3p |
| 3 | 50 | m | CD | A2L3+L4B1 |
| 4 | 25 | f | CD | A1L3+L4B1 |
| 5 | 37 | f | CD | A2L1+L4B3p |
| 7 | 57 | m | UC | E3 |
| 8 | 26 | m | UC | E3 |
| 10 | 26 | m | UC | E3 |
| 13 | 22 | m | CD | A1L3+L4B2 |
| 14 | 55 | m | CD | A2L1B2 |
| 18 | 55 | m | UC | E3 |
| 21 | 66 | m | UC | E2 |
| 22 | 55 | m | UC | E3 |
| 24 | 32 | f | UC | E2 |
| 25 | 57 | m | UC | E3 |
| 26 | 52 | m | UC | E2 |
| 27 | 47 | m | UC | E3 |
| 28 | 49 | f | UC | E2 |
| 29 | 38 | m | CD | A2L3+L4B2p |
| 30 | 44 | f | CD | A2L3B2 |
| 31 | 23 | m | CD | A2L3+L4B2 |
| 32 | 53 | m | CD | A2L3B1 |
| 33 | 55 | m | CD | A3L3+L4B3p |

**Supplementary Table for Figure 3f,g - Immunohistochemistry**

| **ID No.** | **Age** | **Gender** | **Disease** | **Montreal** |
| --- | --- | --- | --- | --- |
| 1 | 42 | f | CD | A2L3B1 |
| 4 | 25 | f | CD | A1L3+L4B1 |
| 6 | 63 | f | CD | A3L3B1 |
| 7 | 57 | m | UC | E3 |
| 8 | 26 | m | UC | E3 |
| 11 | 40 | f | CD | A1L1B2 |
| 12 | 31 | f | CD | A2L3+L4B1 |
| 13 | 22 | m | CD | A1L3+L4B2 |
| 18 | 55 | m | UC | E3 |
| 24 | 32 | f | UC | E2 |
| 25 | 57 | m | UC | E3 |
| 29 | 38 | m | CD | A2L3+L4B2p |
| 34 | 30 | f | CD | A2L3B2B3p |
| 35 | 40 | m | UC | E3 |
| 36 | 35 | f | UC | E3 |
